# Lemierre syndrome: A case report and literature review on atypical presentation

**DOI:** 10.1097/MD.0000000000042823

**Published:** 2025-06-06

**Authors:** Daniel Belay Agonafir, Abraraw Erkie Diress, Aboker Ali Saleh, Chala Jiru Dechasse, Dereje kebede Shane

**Affiliations:** a Department of Internal Medicine, School of Medicine, College of Medicine and Health Sciences, Wachemo University, Hosanna, Ethiopia.

**Keywords:** atypical presentation, case report, ethiopia, internal jugular vein thrombophlebitis, Lemierre syndrome

## Abstract

**Rationale:**

Lemierre syndrome (LS) is a rare but serious condition characterized by septic thrombophlebitis of the internal jugular vein (IJV), often secondary to infections in the head and neck region. It typically begins with an oropharyngeal infection, which may or may not be followed by distant septic emboli, most commonly affecting the lungs. The condition is usually caused by members of the normal oropharyngeal flora, with the anaerobic bacterium Fusobacterium necrophorum being the most prevalent pathogen. Timely diagnosis and antibiotic treatment are essential to prevent adverse outcomes. Clinical circumstances dictate the necessity for therapeutic anticoagulation and surgical intervention. LS can present atypically in terms of the antecedent condition, the etiological agent, the affected vein, and the distant manifestations. Despite the existence of a characteristic clinical presentation, many clinicians remain unaware of this rare condition, which can lead to delayed diagnosis and potentially fatal consequences.

**Patient concerns:**

A 55-year-old man presented with neck pain and swelling that had persisted for 1 week, following 2 weeks of blunt trauma to the head and neck, which resulted in several superficial abrasions due to his poorly managed epilepsy. Imaging studies revealed thrombosis in the left IJV, accompanied by an abscess that extended into the sternocleidomastoid muscle. Gram staining of the specimen obtained from the neck abscess confirmed the presence of Streptococcus bacteria.

**Diagnosis:**

The presence of a thrombus in the IJV following blunt trauma to the head and neck, along with a nearby soft tissue abscess, suggests a diagnosis of LS. However, this case is atypical due to the unusual preceding event of blunt trauma and the identification of a rare pathogen, specifically Streptococcus bacteria.

**Interventions:**

The patient received 2 weeks of intravenous antibiotic treatment with ceftriaxone and metronidazole, followed by 2 additional weeks of oral amoxicillin-clavulanate. Additionally, approximately 100 mL of foul-smelling pus was evacuated from the neck region.

**Outcome:**

The patient has achieved a full recovery.

**Lessons:**

A strong clinical suspicion is essential for diagnosing this uncommon syndrome in patients with a history of head and neck conditions who experience IJV thrombosis or metastatic infections. This research aims to increase awareness of atypical presentations.

## 1. Introduction

Lemierre syndrome (LS), also known as post-anginal sepsis or necrobacillosis, is a rare clinical condition first identified in 1936 by André Lemierre through a study involving 20 cases.^[1]^ This syndrome is characterized by septic thrombophlebitis of the internal jugular vein (IJV), which occurs as a complication of infections in the head and neck region, often initiated by oropharyngeal infections, with or without subsequent distant septic emboli.^[2]^ A prospective study conducted in Denmark between 1998 and 2001 reported an annual incidence of 3 to 6 cases of LS per million individuals, with a notably higher incidence of 14.4 cases per million among those aged 14 to 24 years. The condition is more prevalent in males, with a male-to-female ratio of 2:1.^[3]^ Although the mortality rate associated with LS has decreased from 90% in the pre-antibiotic era to between 4% and 10% in the current antibiotic era, it continues to result in significant morbidity due to its elusive presentation and the growing issue of antibiotic resistance.^[4–7]^

In sub-Saharan Africa, there have been few reported cases of LS.^[5,8,9]^ We present a case involving a 55-year-old male who was diagnosed with LS at Wachemo University Nigist Eleni Mohammed Memorial Comprehensive Specialized Hospital in Ethiopia.

## 2. Case report

A 55-year-old male with a known history of epilepsy for the past 4 years, who has been off his oral medication for the last 3 years, presented to the emergency department with a left lateral neck swelling that had developed over the past week. Initially small, the swelling progressively increased in size and was accompanied by unilateral neck pain and a high-grade intermittent fever of the same duration. Two weeks before the onset of these symptoms, the patient experienced a fall onto his left side due to abnormal body movements characterized by frequent episodes of limb extension and flexion, upward rolling of the eyes, and drooling, lasting approximately 5 minutes without regaining consciousness. He did not seek medical attention following this incident. The patient has a history of pulmonary tuberculosis treatment 2 years ago but reported no other complaints, including cough, dyspnea, chest pain, headache, sore throat, dental pain, nasal congestion or discharge, or any gastrointestinal or urinary issues. He has no known history of other medical conditions or substance abuse.

During the physical examination, the patient exhibited tachycardia, with a heart rate of 128 beats per minute, and a fever, as indicated by an axillary temperature of 38.5°C. Examination of the head and neck revealed a warm, tender mass measuring approximately 3 cm by 3 cm, located laterally to the left sternocleidomastoid muscle. Additionally, there was a healed wound measuring 2 cm by 1 cm on the forehead and left cheek. All other system examinations yielded normal results.

Upon investigation, the complete blood count revealed leukocytosis (14,000/µL, with 75% neutrophils), normal hemoglobin levels (13 g/dL), thrombocytosis (platelet count of 470 × 10^9^/L), and an elevated erythrocyte sedimentation rate (88 mm/hour). Blood glucose levels were within normal limits (random blood sugar of 141 mg/dL), HIV serology tests returned negative results, and both renal and liver function tests were normal.

**Figure 1.**
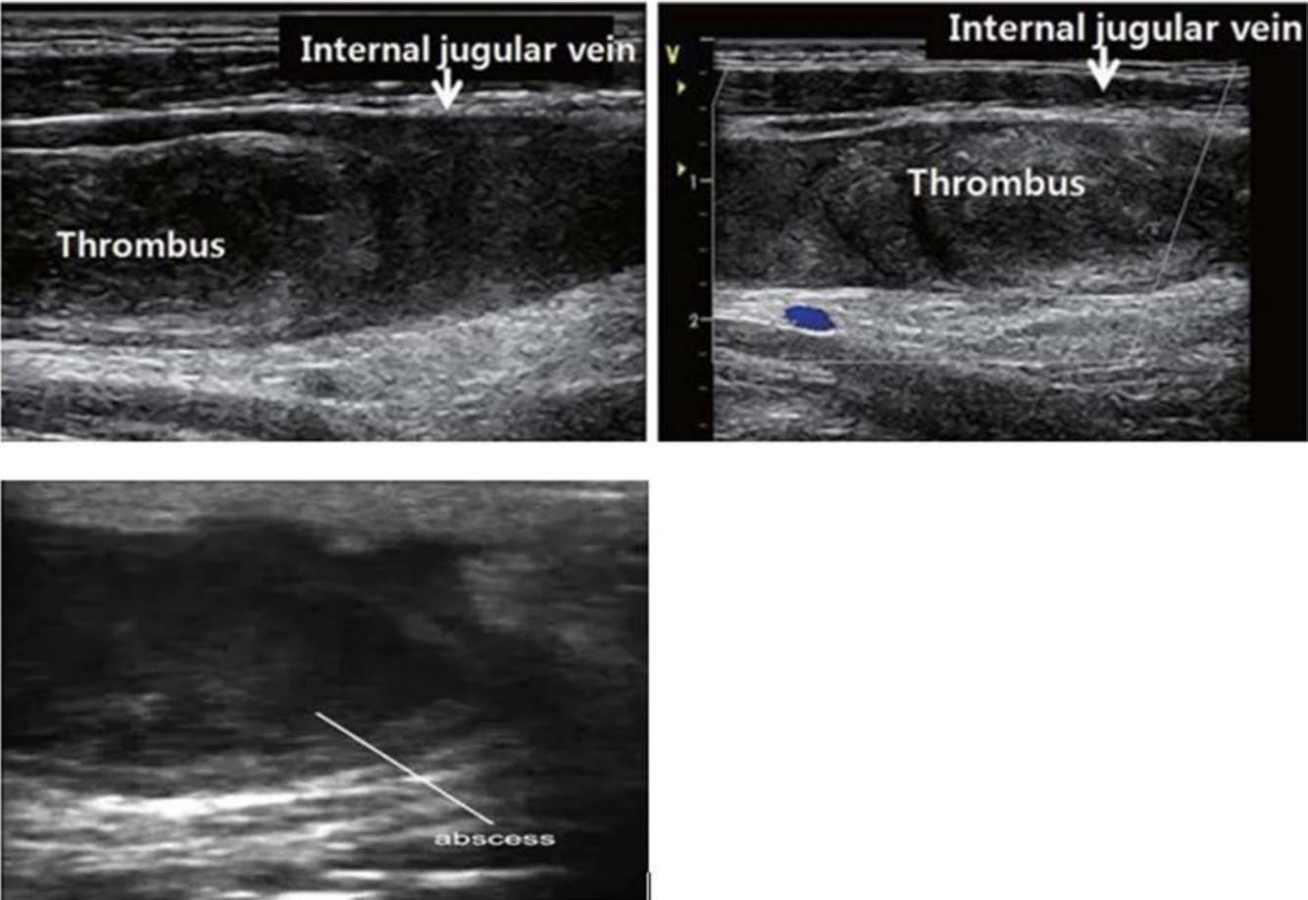
The longitudinal image of the left neck ultrasound shows thrombosis of the left IJV with partial obstruction of the lumen, color flow mapping shows no flow. In addition, 3.9 cm by 2.6 cm left anterolateral neck abscess posterolateral to sternocleidomastoid muscle is seen. IJV = internal jugular vein.

**Figure 2.**
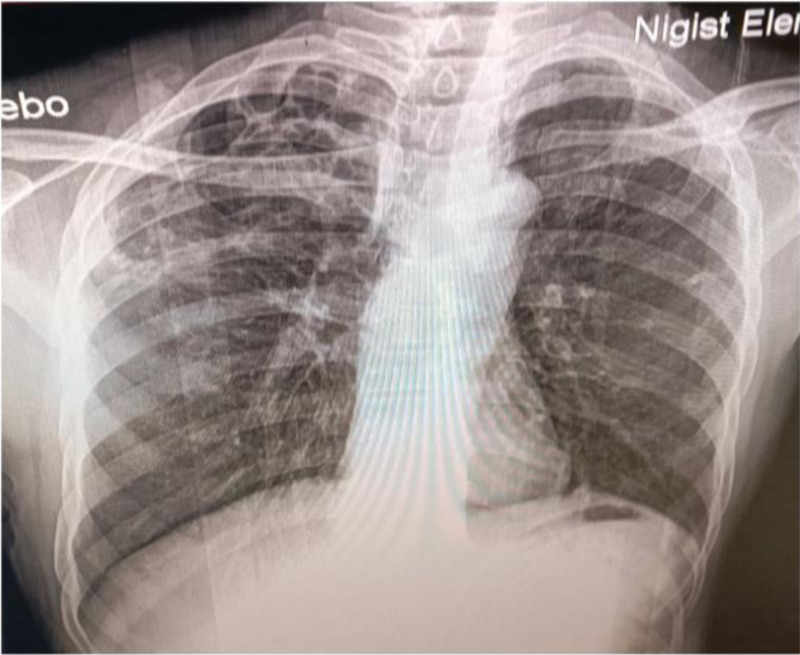
A chest x-ray shows right upper lung zone fibrotic change with no evidence of septic emboli to lung.

A neck Doppler ultrasound was performed (Fig. 1), revealing a hypoechoic filling in the lumen of the left IJV with no detectable flow. Additionally, a heterogeneous hypoechoic collection measuring 3.9 cm by 2.6 cm was identified in the left anterolateral neck, located posterolateral to the left sternocleidomastoid muscle. These findings led to a diagnosis of acute thrombosis of the left IJV and an anterolateral neck abscess. Although a neck and chest computed tomography (CT) scan was indicated at this stage, it could not be performed due to resource constraints. A chest X-ray was subsequently conducted (Fig. 2), which indicated fibrotic changes in the right upper lung zone but showed no signs of septic emboli in the lungs. A sample from the neck abscess was collected for Gram staining, which revealed the presence of gram-positive cocci in chains, suggestive of Streptococcus species. A culture was also planned but could not be executed due to resource limitations. A transthoracic echocardiogram was performed, yielding normal results with no evidence of vegetation.

The presence of a thrombus in the IJV, which occurs following a soft tissue injury in the head and neck region accompanied by a nearby soft tissue abscess, supports the diagnosis of LS. However, this case does not conform to the classical presentation of LS due to the atypical preceding condition of blunt trauma to the head and neck and the identification of an uncommon pathogen, specifically Streptococcus bacteria.

The patient was admitted and started on empirical intravenous antibiotic treatment with ceftriaxone and metronidazole. Approximately 100 mL of foul-smelling pus was drained from the left lateral neck region. Daily wound care was provided.

Following a 2-week course of intravenous antibiotics, the patient made a complete recovery and was discharged with a prescription for oral Augmentin (amoxicillin and clavulanate) to continue for an additional 2 weeks.

**Figure 3.**
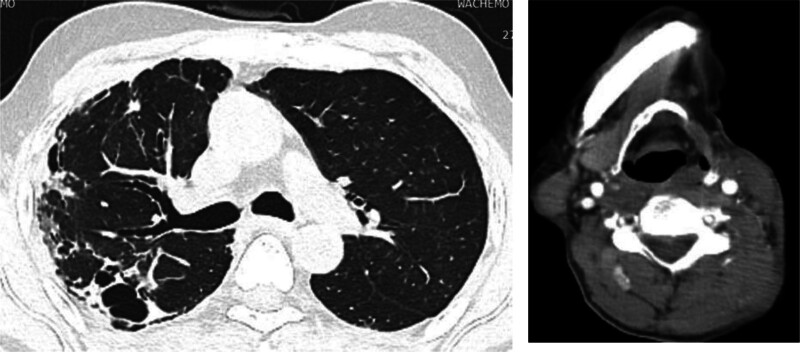
Follow-up neck and chest CT with contrast (done on 12 wk of treatment initiation) shows right upper lobe fibro bronchiectasis likely post tuberculosis with resolution of left internal jugular vein thrombosis. CT = computed tomography.

At the follow-up appointments conducted 2, 6, and 12 weeks after discharge, the patient remained asymptomatic. During this period, a neck and chest CT scan was performed, revealing right upper lobe bronchiectasis likely resulting from previous tuberculosis and the resolution of left IJV thrombosis (Fig. 3).

## 3. Discussion

Lemierre syndrome typically begins with a preceding primary infection in the head and neck region, most commonly with oropharyngeal infections that affect the palatine tonsils or peritonsillar tissues.^[2,7]^ Atypical primary sources of infection may include odontogenic infections, mastoiditis, otitis media, sinusitis, and parotitis.^[10–12]^ In some cases, patients with LS may not present with an antecedent infection^[13]^ or may have experienced blunt neck trauma as a preceding condition.^[14]^ The primary infection is followed by local invasion into the pharyngeal space and the IJV, leading to septic thrombophlebitis, typically occurring within an interval of 1 to 3 weeks^[2]^ (Table 1).

**Table 1:** Literature review on atypical presentation of LS: examples of recently documented case reports.

Author (year)	Antecedent condition	Presenting symptoms	Need for anticoagulation
		Affected vein	
Pathogens	Metastatic infections	Need for surgical procedures
Davis et al, 2024^[11]^	Odontogenic infection	Neck pain and swelling, caries and tooth radix	Yes
		IJV	
	–	None	No
Al-Mashdali and Al-Warqi, 2022^[9]^	acute otitis media	Fever, vomiting, right ear pain	No
		IJV	
	*Streptococcus pneumoniae*	None	No
Denesopolis et al, 2020^[13]^	Blunt cervical trauma	Left neck and shoulder pain	No
		IJV	
	Fusobacterium necrophorum, Staph epidermidis, Methicillin-resistant staph aureus	Disseminated intravascular coagulation	En bloc resection of IJV, drainage of abscesses
Siddique et al, 2020^[12]^	None	Altered mental status, right neck pain	Yes
		IJV	
	*Streptococcus pyogenes*	None	Ligation of IJV
Zamora Gonzalez et al, 2019^[10]^	Submandibular inflammation	Fever, diaphoresis and right submandibular edema	Yes
		Facial vein	
	*Methicillin-resistant Staphylococcus aureus*	Septic emboli to the lungs	No
Laurencet et al, 2019^[14]^	None	Lumbar pain and fever	Yes
		Iliac veins	
	*Fusobacterium nucleatum*	Pneumonia, pleural effusion, spondylodiscitis	Drainage of abscesses
Suzuki et al, 2022^[15]^	None	External jugular vein	Yes
	*Streptococcus intermedius*	Lung abscesses	Thoracic drainage

IJV = internal jugular vein, LS = Lemierre syndrome.

In this case report, the patient developed LS after 2 weeks of sustaining blunt head and neck trauma from a fall, which was attributed to poorly controlled epilepsy.

Members of the normal oropharyngeal flora usually cause LS. The most common causative bacterium is *Fusobacterium necrophorum, an* anaerobic commensal gram-negative bacillus of the oral flora.^[2,7]^ However, atypically other bacteria including other *Fusobacterium* species (e.g., *Fusobacterium nucleatum*), Streptococci, *Staphylococcus aureus*, Bacteroides, Lactobacillus, Enterobacteriaceae and *Eikenella corrodens* have been reported.^[4–6,11,15]^

In this case report, Streptococcus is the microorganism associated with to the development of LS. Similar case reports of patients who developed LS due to Streptococcus have been published.^[10,13,16]^ These reports illustrate that the causal agents may differ from those traditionally described.

Clinical manifestations of LS include fever, sore throat, dysphagia, trismus, exudative tonsillitis, neck pain, tenderness, swelling, and/or induration.^[2,7]^ The IJV is typically affected; however, thrombosis of other veins, such as the facial vein,^[11]^ iliac vein,^[15]^ and external jugular vein,^[16]^ has been reported in atypical cases. Pulmonary complications are common, with lung lesions often presenting as necrotic cavitary lesions due to septic pulmonary emboli. In 2 reports, this finding was observed in 97 percent of cases.^[17,18]^ Other sites may also affected by septic embolization, leading to conditions such as septic arthritis in large joints, soft tissue abscesses, and abscesses in the kidneys, spleen, and liver. Additional complications can include endocarditis, pericarditis, and arterial complications such as mycotic carotid pseudoaneurysms, as well as central nervous system complications, which may encompass meningitis, brain abscesses, subdural empyemas, epidural abscess, septic emboli, cerebral venous thrombosis and strokes.^[18–21]^

In this case report, the clinical manifestations are characteristic of septic thrombophlebitis of the IJV; however, our patient did not experience metastatic complications, like other reported cases.^[10,12,13]^

The diagnosis of LS can be established when radiographic imaging reveals an IJV thrombus, in conjunction with culture results indicating the presence of *F. necrophorum* or other implicated pathogen. Organisms may be isolated from the throat, the blood, and from metastatic infection sites. A CT scan of the neck and chest with contrast allows evaluation of the IJV for filling defects or thrombus; it also facilitates evaluation for lung involvement such as pulmonary emboli and abscesses. Ultrasonography of the neck is an alternative modality for detection of IJV thrombus.^[7,17]^

In this case report, the diagnosis of LS was established through neck ultrasound evidence of IJV thrombosis and the identification of Streptococcus bacteria from a specimen obtained from a neck abscess.

Treatment of LS includes appropriate antibiotic therapy, and consideration of the need for surgical intervention, and anticoagulation. Empiric antibiotic therapy should target *F. necrophorum* and oral streptococci (include Piperacillin-tazobactam, carbapenem, Ceftriaxone plus metronidazole).^[7,17]^ Antibiotics should be tailored accordingly to culture and susceptibility data when available. Clinical circumstances should guide the duration of antibiotic therapy. An antibiotic duration of at least 4 weeks is generally favored, including at least 2 weeks of intravenous therapy.^[3]^

Most patients with LS recovered well without therapeutic anticoagulation. It is reserved for a select group of patients who do not respond to initial antibiotic therapy or if there is a progression of thrombosis or retrograde cavernous or other sinus thrombosis.^[4,22,23]^

The necessity for surgical procedures should be determined by clinical circumstances. If a purulent fluid collection is present, surgical drainage should be considered. If the patient continues to experience persistent sepsis or ongoing septic emboli, surgical ligation or excision of the IJV should be performed.^[24]^

In this case report, the patient fully recovered after 4 weeks of antibiotics treatment (2 weeks intravenously followed by an additional 2 weeks orally) and drainage of a neck abscess, without the need for anticoagulation therapy.

## 4. Conclusions

Lemierre syndrome is characterized by septic thrombophlebitis of the IJV that typically arises from a preceding head and neck infection, most commonly an oropharyngeal infection. This condition can lead to complications such as septic emboli, primarily affecting the lungs. The presentation of LS can be atypical, varying in terms of the antecedent condition, the causative organism, the specific vein involved, and the distant manifestations. A high level of clinical suspicion is essential to prevent delays in diagnosis, which could result in severe and potentially fatal outcomes.

## Author contributions

**Conceptualization:** Daniel Belay Agonafir.

**Data curation:** Daniel Belay Agonafir, Abraraw Erkie Diress, Aboker Ali Saleh, Chala Jiru Dechasse, Dereje Kebede Shane.

**Investigation:** Daniel Belay Agonafir, Abraraw Erkie Diress, Aboker Ali Saleh, Chala Jiru Dechasse, Dereje Kebede Shane.

**Supervision:** Daniel Belay Agonafir, Abraraw Erkie Diress, Aboker Ali Saleh, Chala Jiru Dechasse, Dereje Kebede Shane.

**Validation:** Daniel Belay Agonafir.

**Writing – original draft:** Daniel Belay Agonafir.

**Writing – review & editing:** Daniel Belay Agonafir, Abraraw Erkie Diress, Aboker Ali Saleh, Chala Jiru Dechasse, Dereje Kebede Shane.
